# The Interplay Between Trace Elements, Morphometry, and Semen Quality in Bucks During the Breeding Season

**DOI:** 10.1002/vms3.71130

**Published:** 2026-08-05

**Authors:** Şükrü GÜNGÖR, Muhammed Enes İNANÇ, Mine HERDOĞAN, Durmuş KAHRAMAN, Hasan Ali ÇAY, Feyza Nur MART, Ömer Gürkan DİLEK, Ramazan YILDIZ, Barış Atalay USLU, Ayhan ATA

**Affiliations:** ^1^ Department of Reproduction and Artificial Insemination Faculty of Veterinary Medicine Burdur Mehmet Akif Ersoy University Burdur Türkiye; ^2^ Department of Reproduction and Artificial Insemination Institute of Health Sciences, Burdur Mehmet Akif Ersoy University Burdur Türkiye; ^3^ Department of Anatomy Faculty of Veterinary Medicine Burdur Mehmet Akif Ersoy University Burdur Türkiye; ^4^ Department of Internal Medicine Faculty of Veterinary Medicine Burdur Mehmet Akif Ersoy University Burdur Türkiye; ^5^ Department of Reproduction and Artificial Insemination Faculty of Veterinary Medicine Cumhuriyet University Sivas Türkiye

**Keywords:** blood serum, Hair buck, Honamli buck, morphometric traits, seminal plasma, spermatological parameters, trace elements

## Abstract

**Background:**

Breed‐related differences in spermatological traits, morphometric characteristics and trace element status may influence reproductive performance in bucks during the breeding season.

**Objectives:**

This study aimed to comparatively evaluate spermatological characteristics, body and testicular morphometric traits, and trace element concentrations in blood serum and seminal plasma of Honamli and Hair bucks during the breeding season.

**Methods:**

A total of 16 bucks (8 Honamli and 8 Hair), aged 2–4 years, were included. Semen samples were collected for spermatological evaluation, including mass activity, volume, pH, motility, concentration, plasma membrane and acrosome integrity (PMAI), viability and mitochondrial membrane potential. Seminal plasma was obtained by centrifugation for biochemical analysis. Blood samples were collected from the jugular vein after semen collection, centrifuged and the serum was separated. Trace element concentrations (Se, Zn, Cu, Ca, Mg, Fe and Co) were measured in blood serum and seminal plasma at the beginning and end of the study. Body, scrotal, and testicular morphometric measurements were also recorded.

**Results:**

Honamli bucks exhibited significantly higher sperm motility (*p < *0.05), sperm concentration, PMAI and mitochondrial membrane potential compared with Hair bucks (*p < *0.001), while no significant differences were observed in semen volume, pH, mass activity or viability (*p* > 0.05). Significant breed‐related differences were found in body morphometric traits, including withers height (*p < *0.01), rump height (*p < *0.001), tail length (*p < *0.001), nose length (*p < *0.001) and ear length (*p < *0.05). Only scrotal circumference differed significantly between breeds (*p < *0.05). In blood, Ca, Mg, Zn, Se and Co showed significant time‐dependent changes (*p < *0.001), while Fe also changed over time (*p < *0.01); breed effects were not significant (*p* > 0.05). Furthermore, Ca increased in both breeds (*p < *0.001), Zn increased in Honamli and Hair (*p < *0.01 and *p < *0.05, respectively), Co decreased in both breeds (*p < *0.01) and Se increased only in Hair (*p < *0.001). Significant time × breed interactions were found for Fe and Cu (*p < *0.05); Fe was higher in Honamli on the first day (*p < *0.05) and decreased only in this breed (*p < *0.001), whereas Cu increased only in Hair (*p < *0.05).

In seminal plasma, Se, Fe and Cu changed over time (*p < *0.001), whereas Ca, Zn, Co and Mg remained unchanged (*p* > 0.05); breed and time × breed effects were not significant for any seminal plasma mineral (all *p* > 0.05). In addition, Se increased in both breeds (Honamli: *p < *0.001; Hair: *p < *0.05), while Fe and Cu decreased in both breeds (Fe: *p < *0.01 and *p < *0.05; Cu: *p < *0.01 and *p < *0.001, respectively).

**Conclusions:**

Honamli and Hair bucks display distinct spermatological, morphometric and trace element profiles during the breeding season. These findings indicate that trace element dynamics strongly influence semen quality, suggesting their potential use as markers for sire selection and targeted reproductive management.

## Graphical abstract

Semen, blood serum and morphometric data were collected from Honamli and Hair bucks. Semen samples were evaluated spermatologically and separated for seminal plasma collection, while blood samples obtained on the first and last collection days were processed for serum and stored at –20°C.

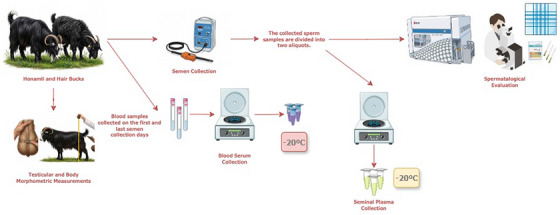

## 1 Introduction

Reproductive performance is a central component of livestock productivity, influencing the continuity of economic yields such as meat, milk and fibre from domestic animals. For sustainable breeding, it is essential to identify males with high fertility potential, as the reproductive efficiency of female counterparts is strongly influenced by the fertilising capacity of sires (Hafez and Hafez 2000). Accurate assessment of male reproductive traits is therefore crucial for improving breeding strategies and maximising reproductive success.

Male fertility in goats is influenced by a complex set of physiological, anatomical and biochemical factors. Among these, spermatological parameters—including sperm motility, concentration, plasma membrane integrity and mitochondrial function—are directly related to fertilising ability and overall semen quality (Asaduzzaman et al. 2022). Testicular morphometric measurements, such as testicular height, scrotal circumference, and scrotal height, are commonly employed as indirect indicators of spermatogenic efficiency and have been linked to semen quality traits in several small ruminant studies (Gibbons et al. 2019; Mohammed et al. 2018). Body morphometric traits, including body length, height and girth measurements, have also been associated with reproductive performance, which may reflect the impact of overall growth and body condition on male fertility (Abdullah et al. 2021; Akounda et al. 2023).

Trace elements are essential for normal physiological processes and have been shown to play important roles in spermatogenesis, sperm function, antioxidant defence and hormonal regulation. Elements such as selenium (Se), zinc (Zn), copper (Cu), calcium (Ca), magnesium (Mg), iron (Fe) and cobalt (Co) act as cofactors in many enzymatic reactions and are involved in membrane stabilisation and energy metabolism of spermatozoa (Aguiar et al. 2012; Barth et al. 2008; Tórtora‐Pérez 2010). Trace elements are essential regulators of biological processes, functioning as enzyme activators or inhibitors, structural components of proteins and modulators of membrane permeability (Vázquez‐Armijo et al. 2011). For instance, Se and Zn have been positively correlated with sperm motility and viability, while deficiencies of key trace elements have been associated with degenerative changes in testicular tissue and impaired reproductive function, often mediated by increased oxidative stress and cellular membrane damage (Colagar et al. 2009; Massányi et al. 2004; Xu et al. 2003; Yuyan et al. 2008).

Although several studies have individually explored morphometric traits, semen quality and trace element status in goats and other ruminants (Devrim et al. 2015; Elmaz, Saatci, Namak, et al. 2012; Inanc et al. 2023), comprehensive evaluations that integrate body and testicular morphometry with trace element profiles in both blood serum and seminal plasma remain limited, particularly in indigenous breed comparisons. To the best of our knowledge, there are no published studies that simultaneously compare spermatological characteristics, blood and seminal plasma trace elements, and morphometric measurements in Honamli and Hair bucks, nor do they analyse the interrelationships among these parameters.

Therefore, this study aimed to comparatively evaluate spermatological characteristics, body and testicular morphometric measurements and trace element concentrations in blood serum and seminal plasma of Honamli and Hair bucks during the breeding season, and to investigate the relationships among these parameters to provide valuable baseline information for reproductive selection, management and targeted breeding strategies in indigenous goat populations.

## 2 Materials and Methods

### 2.1 Animals and Experimental Design

The study was conducted using 16 sexually mature bucks, comprising Honamli (*n = *8) and Hair (*n = *8) breeds, aged between 2 and 4 years. All animals were maintained under identical housing, feeding and management conditions throughout the experimental period. The ration for the buck weighing 90 kg used in the study was prepared according to NRC (2007) goat feeding standards. The ration prepared to meet the animal's energy and protein requirements consisted of 1.00 kg meadow hay and 0.50 kg corn silage as roughage sources, and 0.20 kg barley, 0.10 kg wheat bran and 0.05 kg sunflower meal as concentrate feed sources. In addition, 8–10 g/day of feed salt (NaCl) was added to the ration. Commercial mineral–vitamin premix was not added. Clean drinking water was provided ad libitum. The total dry matter intake of the prepared ration was 1.85 kg. The metabolisable energy value of the ration was calculated as 9.2 MJ/day. The crude protein (CP) content was determined as 160 g/day; the CP ratio in dry matter was calculated as 8.6%, which is consistent with the 8%–10% range. Semen collections were performed once weekly during the breeding season (September–November) at a research facility located in southwestern Türkiye, at an altitude of approximately 1300 m. A total of 80 ejaculates were obtained by electroejaculation, corresponding to five ejaculates (replications) per buck. Immediately after collection, each ejaculate was subjected to routine macroscopic and microscopic evaluation. Individual semen samples were then divided into two aliquots:

- One aliquot was used for spermatological evaluations, and
- The second aliquot was centrifuged for seminal plasma separation.

Blood samples were collected from the vena jugularis immediately after semen collection using gel‐containing vacuum tubes. Blood samples were centrifuged, and serum was separated. Both blood serum and seminal plasma samples were stored at –20°C until trace element analysis.

### 2.2 Spermatological Examinations

#### 2.2.1 Volume and pH

Semen volume was measured directly using graduated collection tubes immediately after ejaculation. Semen pH was assessed using calibrated pH indicator strips.

#### 2.2.2 Mass Activity

Mass activity was evaluated by placing 5 µL of raw semen on a pre‐warmed glass slide and examining sperm movement under a phase‐contrast microscope at 10× magnification. Mass activity was scored subjectively on a scale from 0 to +5 according to sperm density and wave motion patterns (Tırpan 2023).

#### 2.2.3 Motility

Total sperm motility (%) was assessed using a phase‐contrast microscope with a heated platform (37°C–38°C) at 400× magnification. Motility was recorded as the percentage of progressively motile spermatozoa (Güngör et al. 2019).

#### 2.2.4 Flow Cytometric Analysis

Flow cytometric analysis was performed using a CytoFLEX flow cytometer (Beckman Coulter, CA, USA) equipped with a 488 nm laser (50 mW) and emission filters at 525 ± 40 nm, 585 ± 42 nm and 610 ± 20 nm. Spermatozoa were identified based on forward scatter area (FSC‐A) and side scatter area (SSC‐A) characteristics. Doublets were excluded using FSC‐A versus forward scatter height (FSC‐H) gating. Flow cytometric data were analysed using CytExpert software (version 2.3).

##### 2.2.4.1 Spermatozoa Concentration

Semen samples were diluted 1:100 in phosphate‐buffered saline (PBS). An aliquot of 30 µL was loaded into the flow cytometer, and spermatozoa concentration was calculated using CytExpert 2.3 software (Beckman Coulter) (Inanc et al. 2022).

##### 2.2.4.2 Plasma Membrane and Acrosome Integrity (PMAI)

Plasma membrane and acrosome integrity were assessed using the FITC‐PNA/propidium iodide (PI) dual staining method. Briefly, 5µL of FITC‐PNA (100µg/ml; Sigma) and 3µl of PI (2.99 mM; Molecular Probes) were added to 492µl of PBS. Semen samples were diluted to a final concentration of 5 × 10^6^ spermatozoa/ml. Semen samples were incubated at 37°C for 15 min in the dark and subsequently analysed by flow cytometry. Spermatozoa negative for PI and FITC‐PNA were classified as having intact plasma membranes and acrosomes (PMAI, %) (Güngör et al. 2019).

##### 2.2.4.3 Mitochondrial Membrane Potential

Mitochondrial membrane potential was evaluated using the JC‐1 staining method. A total of 5µL of JC‐1 dye (0.153 mM) was added to 492µL of PBS. Semen samples were adjusted to a final concentration of 5 × 10^6^ spermatozoa/mL and incubated at 37°C for 15 min in the dark. Following incubation, samples were analysed by flow cytometry, and the percentage of spermatozoa exhibiting high mitochondrial membrane potential (HMMP, %) was recorded (Güngör et al. 2019).

##### 2.2.4.4 Viability Analysis

Sperm viability was determined using the SYBR‐14/PI dual staining technique. Briefly, 5µL of SYBR‐14 and 3µL of PI were added to 492µL of PBS. Semen samples were adjusted to a final concentration of 5 × 10^6^ spermatozoa/mL and incubated at 37°C for 15 min in the dark. After incubation, samples were analysed by flow cytometry, and viable spermatozoa were identified as SYBR‐positive and PI‐negative cells (%) (Inanc et al. 2023).

### 2.3 Biochemical Trace Element Analysis

Blood serum and seminal plasma samples were analysed for selenium (Se), zinc (Zn), copper (Cu), calcium (Ca), magnesium (Mg), iron (Fe) and cobalt (Co). Analyses were performed on samples collected on the first (replication 1) and last (replication 5) semen collection days.

Sample preparation involved digestion with nitric acid (65%) and hydrogen peroxide (30%), followed by elemental determination using an inductively coupled plasma optical emission spectrometer (ICP‐OES; PerkinElmer PE 5300 DV) (Canbay and Bardakçı 2011).

### 2.4 Testicular and Body Morphometric Measurements

All testicular measurements were performed with the animals in the standing position. Testicular height (TH, mm) was measured as the distance from the proximal end of the caput epididymis to the distal end of the cauda epididymis using a digital calliper. Scrotal circumference (SC, cm) was measured by placing a measuring tape around the widest part of the scrotum. Scrotal height (SH, cm) was measured as the vertical distance from the point where the scrotum attaches to the inguinal region to the distal tip of the scrotum. All measurements were recorded to the nearest millimetre (mm) or centimetre (cm), as appropriate (İnanç et al. 2018).

#### 2.4.1 Body Morphometric Measurements

Body morphometric measurements were recorded according to the methodology described by Yakubu (2009) and Akounda et al. (2023). All measurements were taken with the animals standing on a flat surface and recorded in centimetres (cm).

- *Withers height (WH, cm)*: measured as the vertical distance from the ground to the highest point of the withers at the level of the scapular cartilage.
- *Rump height (RH, cm)*: measured as the vertical distance from the ground to the highest point of the pelvic girdle.
- *Rump width (RW, cm)*: measured as the distance between the two tuber coxae.
- *Body length (BL, cm)*: measured diagonally from the lateral tuberosity of the scapula to the pin bone (tuber ischii).
- *Tail length (TL, cm)*: measured from the base of the tail to the distal end of the coccygeal vertebrae.
- *Chest girth (CG, cm)*: measured as the circumference of the body immediately behind the forelimbs.
- *Nose length (NL, cm)*: measured as the distance from the planum nasale to the pretuberantia intercornualis (between the horns).
- *Horn length (HL, cm)*: measured from the base of the horn along the anterior curvature to the horn tip, without pressing the measuring tape into the grooves.
- *Ear length (EL, cm)*: measured from the base of the ear attachment to the distal tip of the ear.

### 2.5 Statistical Analysis

Semen characteristics, as well as testicular and body morphometric traits, were assessed for normality using the Shapiro–Wilk test and for homogeneity of variances using Levene's test. Variables showing a normal distribution were analysed using Student's *t*‐test or one‐way ANOVA, whereas variables that did not meet the assumption of normality were analysed using the Mann–Whitney U or Kruskal–Wallis tests. Duncan's multiple range test was used for post hoc comparisons.

Mineral concentrations measured in blood and seminal plasma were evaluated by repeated‐measures ANOVA, considering time (first day vs. last day) as the repeated factor and breed (Honamli vs. Hair) as the grouping factor. The effects of time, breed and time × breed interaction were assessed for each variable. When significant differences were detected, Bonferroni‐adjusted pairwise comparisons were used for post hoc evaluation.

To further investigate whether the superior semen was more closely associated with mineral profile or with body/testis size‐related traits, an exploratory principal component analysis (PCA) followed by linear regression analysis was performed using the complete‐case subset in which all relevant measurements were available for the same animals (*n = *16). Separate PCAs were conducted for semen quality variables (concentration, motility, viability, plasma membrane and acrosome integrity, and high mitochondrial membrane potential), mineral variables (Se, Zn, Co, Fe, Cu, Ca and Mg) and body/testis size‐related variables (TH, SH, SC, WH, RH, RW, BL and CG). For each variable block, the first principal component (PC1) was extracted and saved as a regression score. These PC1 scores were then used in linear regression models, with semen quality PC1 as the dependent variable and mineral profile PC1 and body/testis size PC1 as explanatory variables. Because the primary aim of this exploratory analysis was to compare the explanatory value of the PCA‐derived mineral and body/testis variable blocks, breed was evaluated in a separate linear regression model using the same semen quality PC1 outcome, rather than being included in the primary component‐based model. All PCA and regression analyses were conducted on a listwise complete‐case basis.

Statistical analyses of all data obtained from the study were performed using the SPSS 22.00 software package, and the graphs used to present the findings were created using GraphPad Prism 10.2.3 software. Statistical significance was set at *p < *0.05.

## 3 Results

### 3.1 Semen Characteristics

**FIGURE 1:**
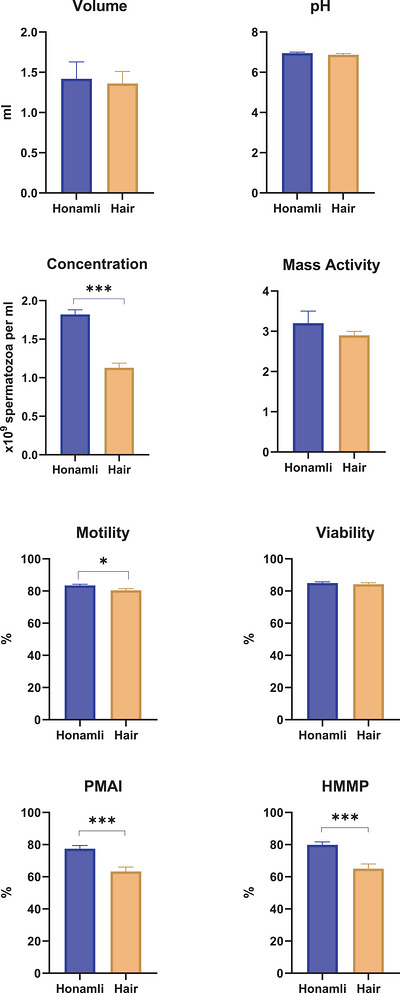
Spermatologic value means of Honamlı and Hair bucks (* *p* < 0.05, ** *p *< 0.01 and *** *p* < 0.001).

Sperm motility (%) was significantly higher in Honamli bucks (83.42 ± 0.82) compared with Hair bucks (80.43 ± 1.02) (*p < *0.05). Similarly, sperm concentration (×10^9^ spermatozoa/ml) was significantly greater in Honamli bucks (1.82 ± 0.06) than in Hair bucks (1.13 ± 0.06) (*p < *0.001). PMAI (%) and HMMP (%) were also significantly higher in Honamli bucks (77.43 ± 2.05 and 79.43 ± 2.05, respectively) compared to Hair bucks (63.33 ± 2.72 and 65.33 ± 2.72), respectively (*p < *0.001). No statistically significant differences were observed between the breeds for semen volume (ml), semen pH, mass activity score or sperm viability (%) (*p* > 0.05). The values obtained from the spermatological evaluation are presented in Figure 1.

### 3.2 Testicular Morphometric Traits

**FIGURE 2:**
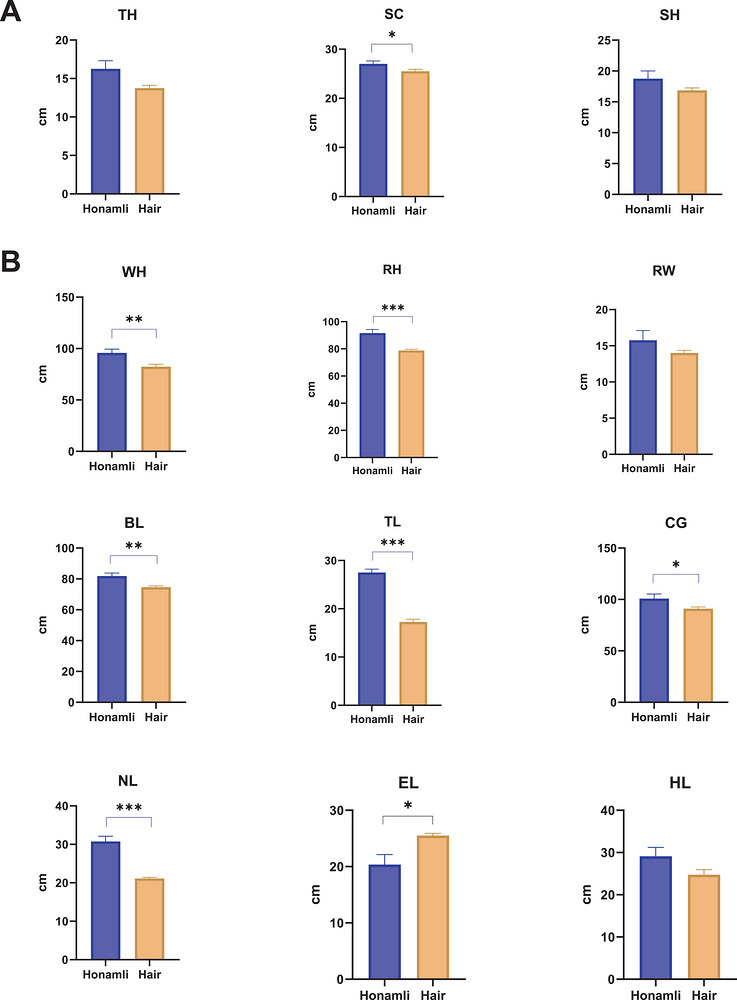
Testicular (A) and Body (B) morphometric measurement value means of Honamli and Hair bucks (* *p* < 0.05, ** *p *< 0.01 and *** *p* < 0.001).

Testicular height (mm) and scrotal height (cm) did not differ significantly between Honamli and Hair bucks (*p* > 0.05). However, scrotal circumference (cm) was higher in Honamli compared to Hair bucks (*p < *0.05). The results of testicular morphometric measurements are shown in Figure 2.

### 3.3 Body Morphometric Traits

Honamli bucks showed significantly greater WH (cm) [95.75 ± 3.74 vs. 82.25 ± 2.47, (*p < *0.01)], RH (cm) [91.62 ± 2.63 vs. 78.75 ± 0.92, (*p < *0.001)], TL (cm) [27.50 ± 0.70 vs. 17.25 ± 0.59, (*p < *0.001)], NL (cm) [30.75 ± 1.37 vs. 21.12 ± 0.29, (*p < *0.001)], and BL (cm) [81.87 ± 1.92 vs. 74.62 ± 0.82, (*p < *0.05)] compared with Hair bucks. In contrast, EL (cm) was significantly higher in Hair bucks (25.50 ± 0.42) than in Honamli bucks (20.37 ± 1.77) (*p < *0.05).

### 3.4 Blood Trace Element Concentrations

In Honamli bucks, blood serum Se, Zn, Mg, and Ca concentrations increased significantly by the end of the study, whereas Co and Fe concentrations decreased (*p < *0.05).

In Hair bucks, blood serum Se, Ca, Zn, and Mg concentrations increased significantly, while Co and Fe concentrations decreased over the study period in both breeds (*p < *0.001). Blood Fe concentrations differed significantly between the two breeds at both the beginning and the end of the study, with higher values observed in Honamli bucks (*p < *0.001).

**FIGURE 3:**
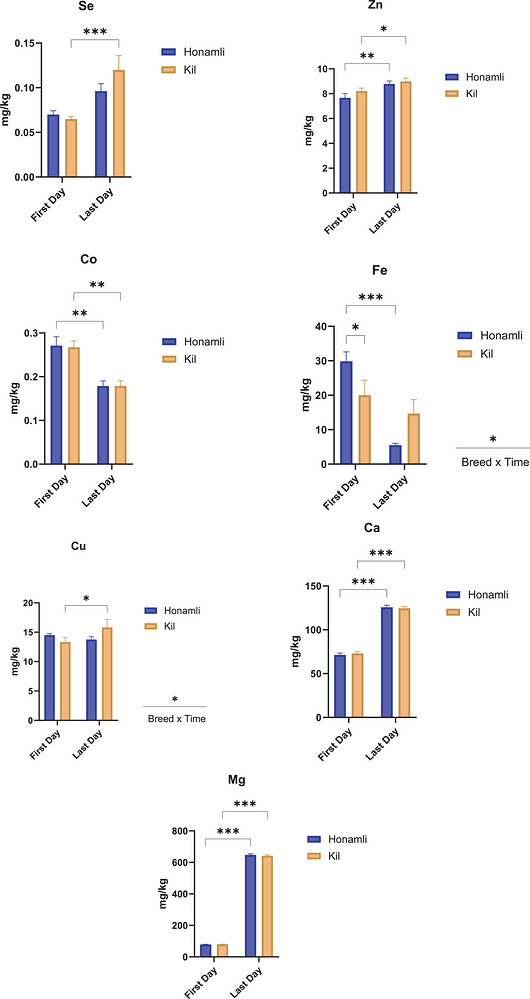
Comparison of blood biochemical parameters of Honamlı and Hair buck (* *p* < 0.05, ** *p *< 0.01 and *** *p* < 0.001). The breed x time interaction was significant (p < 0.05) only for Cu and Fe.

A significant time × breed interaction was observed for blood serum Fe concentrations (*p < *0.01), demonstrating that the rate of decline over the breeding season differed significantly between the breeds. Specifically, Honamli bucks exhibited a markedly more pronounced decrease in Fe levels compared to Hair bucks. Similarly, a significant interaction was detected for blood serum copper (Cu) levels (*p < *0.01), reflecting divergent temporal trends between the two breeds over the course of the study. In contrast, no significant time × breed interactions were found for the remaining blood serum trace elements, including Se, Zn, Ca, Mg and Co (*p* > 0.05). This indicates that the temporal patterns of fluctuation for these elements—whether increasing or decreasing—occurred at a comparable rate and trajectory in both Honamli and Hair bucks. The results of blood serum trace element analyses are illustrated in Figure 3.

### 3.5 Seminal Plasma Trace Element Concentrations

**FIGURE 4:**
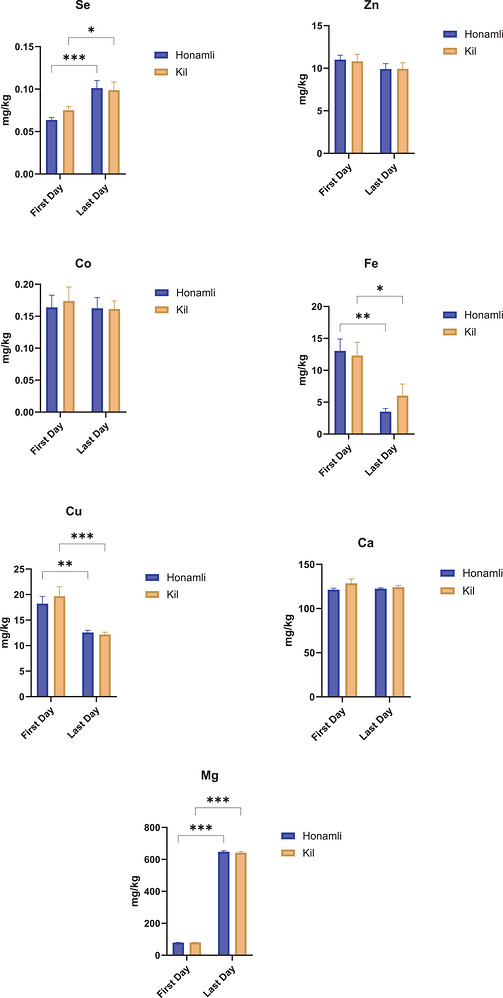
Comparison of biochemical parameters of seminal plasma of Honamli and Hair buck (* *p* < 0.05, ** *p *< 0.01 and *** *p* < 0.001).

In both breeds, regarding the main effect of time, seminal plasma Se levels increased significantly, while Fe and Cu concentrations decreased significantly at the end of the study compared with the beginning (*p < *0.001). Also, seminal plasma Cu and Fe concentrations showed a significant decrease during the study period (*p < *0.001). No significant differences were detected between the two breeds for Zn, Co, Ca and Mg concentrations when comparisons were made at the same sampling time (first or last day) (*p > *0.05). Furthermore, no significant time × breed interaction was detected for any of the trace elements in the seminal plasma (*p > *0.05) as shown in Figure 4.

### 3.6 Linear Regression Analysis

In the complete‐case subset (*n* = 16), the first principal component derived from semen variables explained 69.1% of the total variance and showed positive loadings for concentration, motility, viability, PMAI and HMMP, indicating that it represented an overall semen quality axis. The first principal components derived from mineral variables and body/testis size‐related variables explained 55.7% and 79.7% of the total variance, respectively. In linear regression analysis, mineral profile PC1 was significantly associated with semen quality PC1 in the univariable model (*R* ^2^ = 0.320, *p = *0.022). When body/testis size PC1 was added to the model, the explained variance increased to *R* ^2^ = 0.370; however, this increment was not statistically significant (Δ*R* ^2^ = 0.049, *p = *0.332), and body/testis size PC1 did not contribute significantly to the model (*p = *0.332). In a separate regression model estimated in the same subset, breed also significantly predicted semen quality PC1 (*R* ^2^ = 0.495, *p = *0.002) as shown in Table 1.

**TABLE 1:** Exploratory regression models for semen quality PC1 in the complete‐case subset.

Predictor	Model 1 B (SE)	*p*	Model 2 B (SE)	*p*	Model 3 B (SE)	*p*
Mineral profile PC1	0.566 (0.220)	0.022	0.417 (0.265)	0.139	—	—
Body/testis size PC1	—	—	0.267 (0.265)	0.332	—	—
Breed	—	—	—	—	1.363 (0.368)	0.002
Constant	0.000 (0.213)	1.000	0.000 (0.213)	1.000	−0.681 (0.260)	0.020
R^2^	0.320		0.370		0.495	
Adjusted R^2^	0.272		0.273		0.459	
Overall model p	0.022		0.050		0.002	
ΔR^2^	—		0.049		—	
ΔR^2^ p	—		0.332		—	

*Note*: Dependent variable: semen quality PC1. Model 1 included mineral profile PC1 only. Model 2 additionally included body/testis size PC1. Model 3 was a separate breed‐only model estimated in the same complete‐case subset.

## 4 Discussion

In the present study, the associations between spermatological parameters and trace element concentrations in both blood serum and seminal plasma were evaluated in Honamli and Hair bucks during the breeding season. In addition, the relationships between semen quality and testicular as well as body morphometric measurements were investigated to provide an integrated assessment of male reproductive characteristics in these two indigenous goat breeds.

The descriptive spermatological findings demonstrated that sperm motility, concentration, PMAI and HMMP were significantly higher in Honamli bucks compared to Hair bucks (*p < *0.001), while no significant differences were observed for semen volume, pH, mass activity or sperm viability (*p* > 0.05). These findings are consistent with previous studies conducted in Honamli bucks, which reported comparable values for motility, sperm concentration, PMAI and HMMP during the breeding season (Güngör et al. 2021; Inanc et al. 2023). However, the values observed in the present study were higher than those reported by İnanç et al. (2014), which may be attributed to differences in animal number, management conditions and seasonal effects. Similar to the present findings, semen volume and fresh motility values reported for Gürcü bucks did not differ markedly between seasons, whereas mass activity and sperm concentration were higher during the breeding season (Kulaksız et al. 2019). These variations highlight the combined influence of breed, season and experimental design on spermatological outcomes. The observed motility and sperm concentration values for hairy bucks were consistent with those previously reported by Inanc et al. (2023).

In a subsequent analysis of testicular morphometric measurements, only scrotal circumference had a statistically significant difference between the breeds (*p < *0.05). However, Honamli bucks exhibited significantly greater body measurements, including BL (*p < *0.01), RH, TL, NL (*p < *0.001) and CG (*p < *0.05), while demonstrating shorter EL compared to Hair bucks (*p < *0.05). A review of the extant literature revealed no studies that had concurrently examined testicular and body morphometric evaluations of Honamli and Hair bucks. The present study sought to make a comparison between the morphometric measurement averages of two buck breeds, Honamli and Hair, with a focus on various body dimensions. The findings indicated substantial disparities in multiple parameters, thereby demonstrating the presence of distinct phenotypic characteristics between the breeds. A notable finding was that Honamli bucks exhibited significantly higher values for body length and height, including WH (95.75 cm vs. 82.25 cm; *p < *0.01), RH (91.62 cm vs. 78.75 cm; *p < *0.001) and BL (81.87 cm vs. 74.62 cm; *p < *0.01). These findings suggest a generally larger body size in the Honamli bucks. These differences may result from selective breeding practices aimed at improving traits such as meat or milk production, consistent with findings by Alawiansyah et al. (2020). The phenotypic superiority of the Honamli buck in almost all morphometric parameters (WH, RH, BL and NL) confirms its status as a specialised meat‐type breed with a larger skeletal frame and greater muscle mass potential. In contrast, the Hair goat's significantly longer ears (EL) and smaller frame are classic markers of an indigenous multi‐purpose breed adapted for thermoregulation and survival in rugged terrain. These distinct production purposes, meat production for the Honamli and environmental resilience for the Hair goat, likely dictate their differing trace element requirements. The significantly higher blood Fe levels observed in Honamli bucks may be attributed to the higher myoglobin demands and oxygen‐transport needs of their larger muscle mass. Furthermore, the observed shifts in Zn and Se during the breeding season may reflect Honamli's higher metabolic cost of maintaining a larger body condition while simultaneously supporting spermatogenesis. While previous genetic studies (Korkmaz‐Ağaoğlu et al. 2019) did not find specific growth‐related polymorphisms, our results suggest that the metabolic partitioning of trace elements is breed‐dependent, where the Honamli prioritises mineral mobilisation for rapid growth and meat traits, whereas the Hair goat maintains a more conservative mineral profile suited for long‐term survival and fibre production.

Subsequent comparisons revealed that TL (27.50 cm vs. 17.25 cm; *p < *0.001) and NL (30.75 cm vs. 21.12 cm; *p < *0.001) measurements were also significantly greater in Honamli bucks. Such variations are likely indicative of breed‐specific selection histories or ecological adaptations, as evidenced by the findings of Akounda et al. (2023). In contrast, measurements such as RW and HL exhibited no substantial disparities between the breeds, suggesting that certain morphological traits, possibly associated with reproductive or metabolic functions, may be less impacted by selective breeding. For instance, the similarity in RW (15.75 cm vs. 14.00 cm; *p > *0.05) suggests comparable girth measurements across both breeds. A notable finding was the elevated EL measurement in Hair bucks (25.50 cm) in comparison to Honamli bucks (20.37 cm; *p < *0.05), suggesting potential disparities in fat distribution or body condition. Similarly, the longer tail and nose measurements observed in Honamli bucks may reflect breed‐specific adaptation and selection history, as reported for other goat populations (Akounda et al. 2023). In contrast, the significantly greater ear length observed in Hair bucks may be associated with thermoregulation, body condition or fat distribution, characteristics often observed in native breeds adapted to diverse environmental conditions. These morphometric differences underscore the importance of breed‐specific evaluations when designing breeding and conservation strategies, particularly for indigenous breeds such as the Hair goat, which is valued for its adaptability and resilience (Getaneh et al. 2023).

These observations are corroborated by the findings of previous breed‐specific studies. For instance, body morphometric measurements for Honamli bucks were consistent with prior findings (Elmaz, Saatci, Dag et al. 2012), and comparable morphometric profiles were documented for Hair bucks (Elmaz et al. 2016). Testicular dimensions have been demonstrated to be positively associated with body size and semen quality (Akpa, Ambali, and Suleiman 2013a; Ambali et al. 2013), thereby underscoring the value of such measurements as indicators of reproductive performance. However, investigations into genetic polymorphisms related to growth have not demonstrated significant associations with morphometric traits in either breed (Korkmaz‐Ağaoğlu et al. 2019), suggesting that environmental factors and selective practices may play a more dominant role in shaping body conformation.

Although semen can be collected from bucks throughout the year, reproductive efficiency peaks during the breeding season, and trace element concentrations have been shown to vary markedly between breeding and non‐breeding periods and among breeds. Previous studies reported higher concentrations of zinc in seminal plasma compared to blood serum and increased seminal plasma selenium and zinc levels during the breeding season (Bülbül 2019; Dhami et al. 2001). Also, the significant increase in serum Se and Zn concentrations toward the end of the breeding season (November) suggests a physiological mobilisation of these elements to support sustained spermatogenesis and antioxidant defence during peak reproductive activity. Conversely, the observed decline in Co and Fe may indicate a higher rate of metabolic utilisation or a shift in homeostatic priority as the breeding season progressed. These findings emphasise that trace element profiles are not static but fluctuate in response to the physiological cost of the breeding season in Mediterranean goat breeds.

In the present study, no significant interbreed differences were observed in blood serum Cu concentrations (*p > *0.05). However, a significant decrease in seminal plasma Cu levels was detected in both breeds at the beginning and at the end of the study. Cu is an essential trace element involved in numerous enzymatic systems, cellular respiration, connective tissue formation and antioxidant defence (McDowell et al. 2007; Minatel and Carfagnini 2000). The observed reduction in seminal plasma copper levels during the breeding season may reflect increased utilisation associated with repeated ejaculation and heightened metabolic activity, potentially influencing semen quality.

The exploratory PCA‐based regression analysis performed in the complete‐case subset showed that semen quality was significantly associated with mineral profile in the univariable model. In contrast, adding the body/testis size‐related component resulted in only a small increase in explained variance, which was not statistically significant. These findings indicate that, within the shared subset, variation in semen quality was more closely associated with mineral profile than with the general body/testis size axis derived from morphometric measurements. In a separate regression model estimated in the same subset, breed also significantly predicted semen quality PC1. However, because breed was evaluated separately rather than within the same primary regression model, the present analysis does not allow a direct conclusion as to whether the breed effect was mediated by mineral profile or by body/testis size‐related traits. Therefore, the current findings should be interpreted as showing parallel associations rather than definitive independent explanatory pathways.

## 5 Conclusion

In conclusion, the present study provides a comprehensive comparative evaluation of spermatological characteristics, body and testicular morphometrics, and trace element profiles in blood serum and seminal plasma of Honamli and Hair bucks during the breeding season. Honamli bucks exhibited superior semen quality, characterized by higher sperm motility, concentration, plasma membrane and acrosome integrity, and mitochondrial membrane potential, along with greater scrotal circumference compared with Hair bucks. Distinct breed‐related differences were identified in body morphometric measurements, reflecting divergent phenotypic characteristics likely shaped by selective breeding practices and production purposes. Moreover, dynamic changes in trace element concentrations and their associations with spermatological parameters highlight the importance of mineral status in male reproductive performance. Overall, these findings provide valuable baseline data for Honamli and Hair bucks and emphasize that semen quality differences between breeds may be more closely related to functional and biochemical factors than to testicular size alone. The results may contribute to improved buck selection, reproductive management, and the development of targeted breeding strategies under tropical and subtropical production conditions.

## Author Contributions

**Şükrü Güngör** : writing – review and editing, supervision, project administration, methodology, data curation, conceptualization. **Muhammed Enes Inanç** : supervision, writing – original draft, visualization, validation, resources, formal analysis, writing – review and editing. **Mine Herdoğan** : spermatological examination, data collection, formal analysis. **Durmuş Kahraman** : animal handling, spermatological examination, resources. **Hasan Ali Çay** : formal analysis, resources. **Feyza Nur Mart**: formal analysis. **Ömer Gürkan Dilek** : morphometric measurements, writing – original draft, resources. **Ramazan Yildiz** : trace element examination and evaluations, editing and formal analysis. **Barış Atalay Uslu** : methodology, data curation, conceptualization. **Ayhan Ata** : review and editing.

## Funding

This research was supported by Burdur Mehmet Akif Ersoy University Scientific Research Projects Coordination, Project No. 0727‐MP‐21/2017K12‐41003.

## Ethics Statement

All procedures involving animals in the present study were conducted in accordance with ethical standards and were approved by the relevant Local Ethics Committee on Animal Research. The full unblinded ethics approval information is provided on the title page.

## Conflicts of Interest

The authors declare no conflicts of interest.

## Data Availability

The datasets generated and/or analysed during the current study are available from the corresponding author upon reasonable request.
